# Multiple factor assessment for determining resting metabolic rate in young adults

**DOI:** 10.1038/s41598-024-62639-2

**Published:** 2024-05-23

**Authors:** Wanqing Zhou, Hong Su, Jiali Tong, Wenwen Du, Bo Wang, Pei Chen, Hua Wan, Ming Zhou

**Affiliations:** 1 Department of Nutrition and Food Hygiene, School of Public Health, Nanjing Medical University, Nanjing, 211166 China; 2 Sir Run Run Hospital, Nanjing Medical University, Nanjing, 211166 China; 3 Department of Nutrition and Food Hygiene, Gusu School, Nanjing Medical University, Suzhou, 215004 China

**Keywords:** Nutrition, Weight management

## Abstract

Existing formulas cannot fully explain the variation of resting metabolic rate (RMR). This study aims to examine potential influencing factors beyond anthropometric measurements and develop more accurate equations using accessible parameters. 324 healthy adults (230 females; 18–32 years old) participated in the study. Height, fat-free mass (FFM), fat mass (FM) and RMR were measured. Menstrual cycle, stress levels, living habits, and frequency of consuming caffeinated foods were collected. Measured RMR were compared with predictive values of the new equations and previous 11 equations. Mean RMR for men and women was 1825.2 ± 248.8 and 1345.1 ± 178.7 kcal/day, respectively. RMR adjusted for FFM^0.66^FM^0.066^ was positively correlated with BMI. The multiple regression model showed that RMR can be predicted in this population with model 1 (with FFM, FM, age, sex and daily sun exposure duration) or model 2 (with weight and height replacing FFM and FM). The accuracy was 75.31% in the population for predictive model 1 and 70.68% for predictive model 2. The new equations had overall improved performance when compared with existing equations. The predictive formula that consider daily sun exposure duration improve RMR prediction in young adults. Additional investigation is required among individuals in the middle-aged and elderly demographic.

## Introduction

Alarming statistics show that the number of people affected by excessive weight has surpassed 2 billion, representing approximately 30% of the world’s population^1^. The balance between total energy expenditure (TEE) and energy intake determines body mass^2^. The accurate determination of TEE is important for establishing dietary intake goals in weight management of normal weight and obese individuals.

TEE is the sum of resting metabolic rate (RMR), physical activity energy expenditure, and the thermic effect of food. RMR accounts for 60–70% of the total daily energy expenditure^3^. Indirect calorimetry (IC) is considered the reference method for determining the RMR by measuring O_2_ consumption and CO_2_ production^4^. However, due to its strict evaluation conditions, high cost, time it requires (30–50 min), and lack of portability, the application of IC is limited.

Consequently, several predictive equations have been developed to estimate RMR as an alternative to directly measuring RMR^5–9^, These equations utilize various anthropometric parameters, including age, body weight, height, sex, and fat-free mass (FFM). Since the majority of RMR predictive equations were proposed decades ago and based on specific individual cohorts with different biological and metabolic characteristics from those of the current population, it is not possible to accurately obtain all individuals’ RMR by using a single standard prediction equation^10^.

What is more, these variables can account for only approximately 70% of the variation in RMR between individuals^11^. A few studies have explored the relationship between vitamin D, stress, physical activity, menstrual cycle, smoking, drinking, bedtime, measuring seasons, diet and RMR^12–18^. These factors may affect resting metabolism and contribute to unexplained variation. To date, no studies have taken these factors into account simultaneously.

Therefore, this study aims to examine potential influencing factors beyond age, body weight, height, sex, and FFM, and develop more accurate equations in a sample of adults using accessible parameters.

## Methods

### Participants

We first used the statistical formula [n = (U_α_ × σ/δ)^2^] to calculate the lowest sample size. In the formula, α was significant level and the value was 0.05, and U value was 1.96; σ represented the standard deviation of RMR, for which we referred to values reported by previous Chinese studies^19^; δ was acceptable error. By the calculation, the lowest number was 276. A cross-sectional study was conducted between May 2022 and October 2023. Participants were recruited through advertisements. Inclusion criteria were college students (including undergraduate students) in Jiangning District. Exclusion criteria included having diseases that could affect the measurement of gas exchange and body metabolism, such as asthma and chronic obstructive pulmonary disease. Before participation, each individual received comprehensive information about the nature and purpose of the study and provided written consent. Informed consent was obtained from all subjects and/or their legal guardian(s). The study was conducted in accordance with the Declaration of Helsinki, and was approved by the Ethics Committee of Nanjing Medical University (Ethical code: 2022-680).

### Questionnaire/demographic data

Demographic data were collected using a questionnaire, completed online by participants the day before the measurement. The responses were carefully reviewed by researchers prior to the actual measurements. The collected information included sex, age, menstrual cycle, health condition, stress levels, physical activity, daily sun exposure duration (less than 15 min/day, 15–30 min/day, 31–45 min/day and more than 45 min/ day), frequency of sun protection (never, sometimes, often and always), bedtime (before 23 p.m., 23–24 p.m. and after 24 p.m.), and the frequency of consuming caffeinated foods. The menstrual phase was reported by each woman. The luteal phase was the 14 days before the next menses. The follicular phase was the time from the first day post-menses to the day before luteal phase. Questions related to the level of physical activity were derived from the Chinese version of the International Physical Activity Questionnaire (IPAQ)^20^. Similarly, short-term and chronic stress were evaluated using a 10-item Chinese version of the Perceived Stress Scale (PSS) and the short version of the Trier Inventory for Chronic Stress (TICS), respectively^21,22^.

### Anthropometric measurements

Participants were instructed to visit the Sir Run Run Hospital (Jiangsu, China) following a minimum 10 h fasting period, during which they refrained from consuming any stimulant substances (e.g., caffeine, tobacco) for at least 4 h. Additionally, participants were required to abstain from engaging in vigorous physical activity for 24 h prior to their visit^23^. All measurements were performed between 8:00 and 10:30 in the morning.

Height was measured in duplicate to the nearest 0.1 cm using an ultrasonic stadiometer (EH201R, Xiangshan, Guangdong, China). All subjects were asked to remove their shoes, socks and any heavy clothing. Body composition was assessed by bioelectrical impedance analysis (MC-780MA, TANITA, Tokyo, Japan). After inputting the gender, age, and height data into the device and applying weight adjustment for clothing, participants were instructed to stand barefoot on the analyzer and grip the handles. The recorded measurements included weight (kg), fat mass (FM, kg), and FFM (kg). Body mass index (BMI) was calculated as body weight (kg) divided by squared height (m^2^). BMI was categorized according to the classification criteria of the World Health Organization.

### RMR measurement and prediction

RMR was assessed using indirect calorimetry (Quark PFT, COSMED, Rome, Italy). On each testing day, a 30 min warm-up time for the system was required. Before the measurement, the researcher performed flowmeter and gas calibrations following the instructions of the manufacturer’s instructions. Participants were asked to stay awake in a supine position with a canopy system after a 20 min rest. Oxygen consumption (VO_2_) and carbon dioxide production (VCO_2_) were measured at 10 s intervals for 15 min, and values from the first 5 min were discarded^24,25^. Data of VCO_2_ and VO_2_ during the first 5 min period with the lowest coefficient of variance (CV) from RMR were averaged and used in the analysis. RMR was calculated using the Weir’s equation^26^. All participants were measured by the same researcher. We excluded RMR data from the analysis if the respiratory quotient (RQ) was outside the expected range (0.70–1) and when the measured RMR was more than ± 3 standard deviations from the mean RMR. Considering that weight is the major determinant of RMR and the components of weight (i.e. FM and FFM) contribute unequally to RMR, RMR indexed to [FFM^0.66^ × FM^0.066^] was calculated to find the association between BMI and RMR^27^. RMR was estimated for each subject using the following predictive equations: Mifflin et al., HB (Harris & Benedict), Owen, Müller, Liu, Yang, Singapore, Xue and Wang equations^5–9,19,28–31^. These equations are either widely used in China or established based on Chinese population data. Müller and Xue equations were further categorized into Müller_W, Müller_BC, Xue_W and Xue_BC according to the different parameters (weight or body composition parameters) they use.

### Statistical analysis

Statistical analyses were performed using SPSS 21. Visual inspection of normal Q–Q plots and skewness/kurtosis values were used to determine whether the parameters were normally distributed. Descriptive data were presented as mean ± SD, median (25th–75th percentile) or n (%) where appropriate.

Univariate associations between potential influencing factors and RMR were explored by simple linear regression to identify predictors for inclusion in multivariate regression modeling with backward selection. Measured and predicted RMR were compared at a group level using a paired t-test. The percentage of participants whose predicted RMR value was within 90–110% of the measured RMR was defined as a precise measurement. Overestimation and underestimation were defined as > 110% and < 90% of measured RMR, respectively, and were reported as percentage of subjects. Bland and Altman plots and the intraclass correlation coefficient (ICC) were used to assess the agreement between the RMR estimated by the proposed equation and the RMR_IC_. The threshold for significance in all tests was set at P < 0.05 (two sided).

## Results

### Population characteristics

A total of 324 participants (94 males and 230 females) were included in this analysis. Table 1 summarizes the demographic, anthropometric, and body composition variables. All subjects were between 18 and 32 years old. Overall, 228 (70.4%) were normal weight, 2 (0.6%) were smokers and 16 (4.9%) were drinkers.

**Table 1:** Demographic, anthropometric, and body composition variables of subjects (n = 324).

Characteristics		Total (n = 324)	Male (n = 94)	Female (n = 230)
Age, years		20.7 ± 2.5	20.8 ± 2.5	20.7 ± 2.6
Height, cm		167.5 ± 8.0	176.2 ± 6.0	164.0 ± 5.6
Weight, kg		59.9 ± 12.5	71.2 ± 13.7	55.3 ± 8.5
FM, kg		14.8 ± 6.2	13.0 ± 7.3	15.5 ± 5.6
FFM, kg		45.1 ± 9.9	58.2 ± 7.8	39.8 ± 3.8
BMI	Under weight	60 (18.5%)	9 (9.6%)	51 (22.2%)
	Normal weight	228 (70.4%)	62 (66.0%)	166 (72.2%)
	Overweight or obesity	36 (11.1%)	23 (24.5%)	13 (5.7%)
Sleeping duration, hours		7.1 ± 0.9	7.1 ± 0.7	7.1 ± 1.0
PSS-10 score		18.0 ± 5.5	17.4 ± 6.0	18.3 ± 5.3
TICS-9 score		23.6 ± 5.5	23.3 ± 6.0	23.7 ± 5.3
Menstrual cycle	Follicular phase	83 (36.1%)	–	83 (36.1%)
	Luteal phase	111 (48.3%)		111 (48.3%)
	Menstrual phase	36 (15.7%)		36 (15.7%)
Smoking	Never smoke	322 (99.4%)	92 (97.9%)	232 (100%)
	Current smoke	2 (0.6%)	2 (2.1%)	0 (0%)
Drinking	Never drink	308 (95.1%)	88 (93.6%)	220 (95.7%)
	Current drink	16 (4.9%)	6 (6.4%)	10 (4.3%)
Bedtime	Before 23 p.m	23 (7.1%)	8 (8.5%)	15 (6.5%)
	23–24 p.m	148 (45.7%)	43 (45.7%)	105 (45.7%)
	After 24 p.m	153 (47.2%)	43 (45.7%)	110 (47.8%)
Daily sun exposure duration	< 15 min	48 (14.8%)	7 (7.4%)	41 (17.8%)
	15–30 min	127 (39.2%)	33 (35.1%)	94 (40.9%)
	31–45 min	79 (24.4%)	29 (30.9%)	50 (21.7%)
	> 45 min	70 (21.6%)	25 (26.6%)	45 (19.6%)
Frequency of sun protection	Never	73 (22.5%)	59 (62.8%)	14 (6.1%)
	Sometimes	106 (32.7%)	26 (27.7%)	80 (34.8%)
	Often	88 (27.2%)	5 (5.3%)	83 (36.1%)
	Always	57 (17.6%)	4 (4.3%)	53 (23.0%)
Vitamin D supplement	Yes	16 (4.9%)	4 (4.3%)	12 (5.2%)
	No	308 (95.1%)	90 (95.7%)	218 (94.8%)
Measuring seasons	Spring	104 (32.1%)	28 (29.8%)	76 (33.0%)
	Summer	106 (32.7%)	37 (39.4%)	69 (30.0%)
	Autumn and winter	114 (35.2%)	29 (30.9%)	85 (37.0%)
Physical activity	Low-level	58 (17.9%)	5 (5.3%)	53 (23.0%)
	Moderate-level	149 (46.0%)	39 (41.5%)	110 (47.8%)
	High-level	117 (36.1%)	50 (53.2%)	67 (29.1%)
Frequency of consuming caffeinated foods, times/year	Tea	5 (0–52)	0 (0–24)	11 (0–52)
	Tea beverage	24 (3–60)	39 (8–104)	24 (2–52)
	Fresh coffee	2 (0–24)	0 (0–21)	6 (0–24)
	Instant coffee	0 (0–12)	0 (0–12)	0 (0–19)
	Coffee drink	0 (0–12)	0 (0–12)	0 (0–12)
	Chocolate products	24 (2–52)	12 (0–36)	24 (8–60)
	Tea with milk	36 (12–52)	14 (2–52)	36 (12–52)
	Cola	12 (0–36)	24 (3–52)	6 (0–24)
	Functional drink	0 (0–0)	0 (0–7)	0 (0–0)

Results are presented as mean ± SD, median (25th–75th percentile) or n (%).

*FM* fat mass, *FFM* fat-free mass, *BMI* body mass index, *PSS-10* Perceived Stress Scale, *TICS-9* Trier Inventory for Chronic Stress.

**Figure 1:**
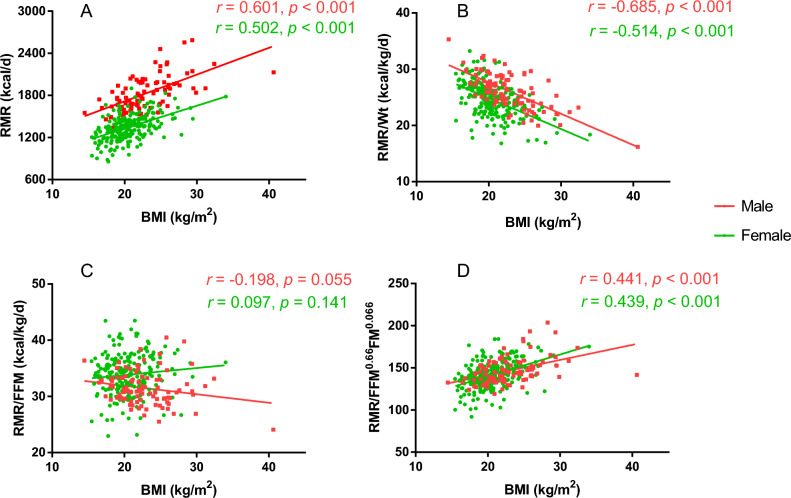
Association between BMI and RMR (**A**), RMR/Wt (**B**), RMR/FFM (**C**) and RMR/FFM^0.66^FM^0.066^ (**D**). *RMR* resting metabolic rate, *Wt* weight, *FM* fat mass, *FFM* fat-free mass, *BMI* body mass index.

Mean RMR for men and women was 1825.2 ± 248.8 and 1345.1 ± 178.7 kcal/day, respectively (Table 2). Males have higher RMR/Wt and RMR/FFM^0.66^FM^0.066^ values, while females have higher RMR/FFM values. RMR and RMR adjusted for FFM^0.66^FM^0.066^ were positively correlated with BMI (Fig. 1). When normalizing RMR by body weight, RMR decreased significantly as BMI increased. After adjusting for FFM, the association was not significant.

**Table 2:** RMR and standardized RMR of different groups of subjects.

	Male	Female	*P*-value
RMR, kcal/d	1825.2 ± 248.8	1345.1 ± 178.7	< 0.001
RMR/Wt, kcal/kg/d	26.1 ± 3.2	24.6 ± 2.9	< 0.001
RMR/FFM, kcal/kg/d	31.5 ± 3.0	33.9 ± 3.5	< 0.001
RMR/FFM^0.66^FM^0.066^	146.6 ± 15.9	141.4 ± 16.2	0.008

*RMR* resting metabolic rate, *Wt* weight, *FM* fat mass, *FFM* fat-free mass, *BMI* body mass index.

### Developing new predictive equations

RMR was selected as the dependent variable, and all variables presented in Table 1 as independent variables. A univariate regression analysis was conducted for each variable (Table 3). The results indicate that, in addition to height, weight, FM, FFM and BMI, daily sun exposure duration, frequency of sun protection, physical activity and the consumption frequency of tea beverages, tea with milk, cola and functional drinks were found to be significant.

**Table 3:** Results of univariate regression analysis.

Characteristics	Coefficients	95% CI	P-value
Age, years	− 9.311	− 22.119, 3.497	0.154
Height, cm	26.701	23.881, 29.521	< 0.001
Weight, kg	18.707	17.115, 20.300	< 0.001
FM, kg	10.460	5.380, 15.539	< 0.001
FFM, kg	25.804	24.132, 27.476	< 0.001
Sleeping duration, hours	− 12.139	− 48.084, 23.806	0.507
PSS-10 score	− 1.091	− 6.982, 4.800	0.716
TICS-9 score	− 1.302	− 7.241, 4.636	0.666
Menstrual cycle	− 7.584	− 41.302, 26.133	0.658
Smoking	118.526	− 88.446, 325.499	0.261
Drinking	34.400	− 40.473, 109.273	0.367
Bedtime	− 4.008	− 56.552, 48.536	0.881
Daily sun exposure duration	72.975	41.108, 104.841	< 0.001
Frequency of sun protection	− 142.445	− 170.172, − 114.718	< 0.001
Vitamin D supplement	25.021	− 124.889, 174.932	0.743
Measuring seasons	− 14.195	− 53.796, 25.407	0.481
Physical activity	101.237	56.982, 145.492	< 0.001
Tea	− 0.235	− 0.668, 0.198	0.286
Tea beverage	0.843	0.373, 1.313	< 0.001
Fresh coffee	− 0.129	− 0.556, 0.298	0.554
Instant coffee	0.013	− 0.323, 0.349	0.939
Coffee drink	0.755	− 0.060, 1.569	0.069
Chocolate products	− 0.359	− 0.788, 0.071	0.102
Tea with milk	− 0.727	− 1.394, -0.060	0.033
Cola	0.866	0.242,1.489	0.007
Functional drink	3.217	1.751, 4.682	< 0.001

*CI* confidence interval, *FM* fat mass, *FFM* fat-free mass, *BMI* body mass index, *PSS-10* Perceived Stress Scale, *TICS-9* Trier Inventory for Chronic Stress.

The significant variables were included in the multiple regression analysis model. Additionally, weight and body composition parameters (FM and FFM) were entered into two separate models. The results are presented below:

$$ \begin{aligned} Model \, 1:{\text{ RMR}}\, & = \,{629}.0{66}\, + \,{1}0{5}.{861}\, \times \,{\text{Sex}} - {12}.{5}0{1}\, \times \,{\text{Age }}\left( {{\text{years}}} \right)\,+ \,{6}.{4}0{3}\, \times \,{\text{FM}}\,  \\ & \quad  + \,{2}0.{838}\, \times \,{\text{FFM}}\, + \,{19}.{271}\, \times \,{\text{E }}({\text{R}}^{{2}} \, = \,0.{772}), \end{aligned} $$

$$ \begin{aligned} Model \, 2:{\text{ RMR}}\, & = \,{58}.{399}\, + \,{4}.{838}\, \times \,{\text{Height }}\left( {{\text{cm}}} \right) - {9}.{732}\, \times \,{\text{Age }}\left( {{\text{years}}} \right)\, + \,{228}.{826}\, \times \,{\text{Sex}}\, \\ & \quad + \,{2}0.{731}\, \times \,{\text{E}}\, + \,{11}.{66}0\, \times \,{\text{Weight }}\left( {{\text{kg}}} \right) \, ({\text{R}}^{{2}} \, = \,0.{764}), \end{aligned} $$

$$ \begin{aligned} ({\text{Sex}}:{\text{ male}}\, & = \,{1},{\text{ female}}\, = \,0;{\text{ E}}:{\text{ daily sun exposure duration}},\, < \,{15}\; {\text{min}}/{\text{d}}\, \\ &= \,{1},{ 15}{-}{3}0 \;{\text{min}}/{\text{d}}\, = \,{2},{ 31}{-}{45} \;{\text{min}}/{\text{d}}\, = \,{3},\, > \,{45}\; {\text{min}}/{\text{d}}\, = \,{4}). \end{aligned} $$

### Validation of predictive equations

The validation results of the newly developed equation are presented in Table 4. Most of the other equations underestimated RMR, except for the equations by HB and Xue. The newly developed predictive formulas showed the lowest RMSE value of 141.63 kcal (*Model 1*) and the highest ICC (0.871). The Xue equation, which integrated body composition parameters, displayed the highest bias.

**Table 4:** Evaluation of new and selected predictive equations.

Equations	pRMR, kcal/day	Bias, %	ICC	RMSE, kcal/day
Model 1	1484.4 ± 260.7	1.0	0.871	141.63
Model 2	1485.5 ± 259.4	1.0	0.867	143.96
Mifflin	1430.6 ± 220.1∗	− 2.4	0.815	160.52
HB	1505.3 ± 222.9∗	2.9	0.827	154.41
Owen	1311.7 ± 208.6∗	− 10.5	0.655	235.96
Müller_W	1438.0 ± 222.7∗	− 1.8	0.817	159.74
Müller_BC	1419.3 ± 218.0∗	− 3.1	0.803	166.10
Liu	1377.3 ± 233.0∗	− 6.2	0.771	187.85
Yang	1381.6 ± 308.8∗	− 6.6	0.791	201.24
Singapore	1278.7 ± 222.0∗	− 13.0	0.630	257.83
Xue_W	1647.4 ± 255.9∗	12.4	0.729	220.83
Xue_BC	1694.6 ± 262.3∗	15.7	0.671	257.20
Wang	1324.6 ± 230.2∗	− 9.8	0.698	224.31

Average RMR measured with indirect calorimetry = 1484.4 ± 296.8 kcal/day.

*pRMR* predicted resting metabolic rate, *ICC* intraclass correlation coefficient, *RMSE* root mean square error.

*P < 0.05, pRMR versus mRMR.

**Figure 2:**
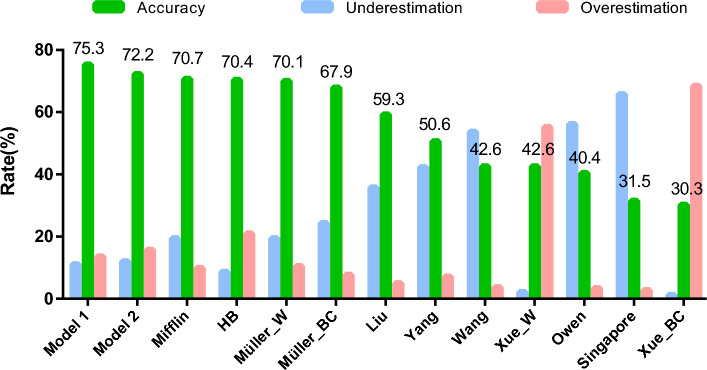
Accuracy of prediction equations for measurements of resting metabolic rate within ± 10%.

The percentages of underestimated, accurate, and overestimated subjects for each equation are presented in Fig. 2. Among the thirteen equations, the highest accuracy rate was produced by the newly developed equation (75.31%). Other equations demonstrating relatively high accuracy rates included Mifflin, HB, and Müller_W (70.68%, 70.37% and 70.06%, respectively); however, they exhibited elevated rates of underestimation or overestimation. The overestimation rate of HB was 20.99%, and the underestimation rates of Mifflin and Müller_W were both 19.44%. It is worth highlighting that the Singapore equation yielded the highest underestimation rate (65.74%). The Xue_BC equation provided the lowest accuracy rate (30.25%) and the highest overestimation rate (68.52%).

**Figure 3:**
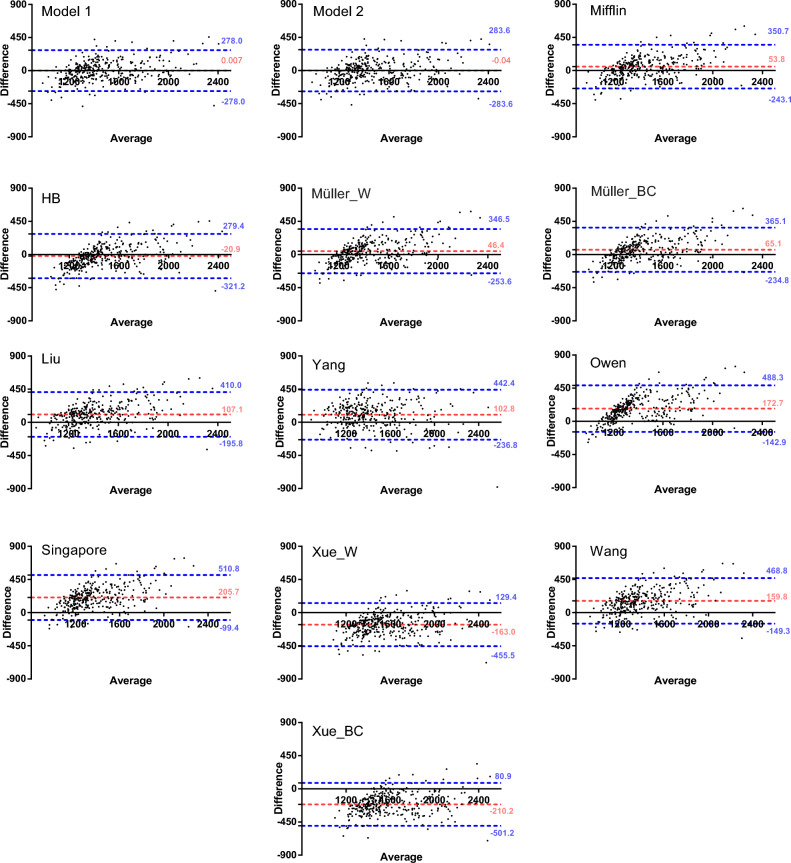
Bland–Altman plots for selected resting metabolic rate (RMR) predictive equations. The red dashed lines represent the mean difference (BIAS) between predicted and measured RMR. The upper and lower blue dashed lines represent the 95% limits of agreement. The X-axis represents the mean of mRMR and pRMR. The Y-axis represents the difference between mRMR and pRMR.

Lastly, the Bland–Altman plots were used to assess the agreement between pRMR and mRMR. Figure 3 shows that the new equations had the best agreement. Our mean bias was closest to X-axis, while Xue_BC’s value was − 210.2 kcal/day away from X-axis. The 95% limits of agreement for the other equations were wider, with the Yang equation exhibiting the largest values (− 236.8 to 442.4 kcal/day). New equations appear to offer a more accurate prediction of RMR.

## Discussion

The present study generated two models that provide the best prediction of RMR. *Model 1* includes age (years), sex, FFM (kg), FM (kg) and daily sun exposure duration as predictors, with an R^2^ value of 0.772. Considering that body composition parameters are not always available, *model 2* was devised by substituting body weight for FM and FFM. Since the previous equations tended to either overestimate or underestimate RMR, they cannot accurately predict the RMR of Chinese young adults.

In several cases, researchers divide RMR by the body weight of individuals to standardize possible variations due to body size^32^. However, this approach has been a subject of debate over the years, due to the unequal contributions of FM and FFM to RMR. FFM has been established as more metabolically active than fat mass. Thus, normalization of RMR by FFM is widely used and considered superior to division by body weight. Nevertheless, it is crucial to recognize that adipose tissue has a mass-specific metabolic rate of 4.5 kcal/kg/day^33^. Using FFM alone for normalization may be inadequate in light of this fact. Francois Haddad et al.^27^ indicated that RMR indexed to [FFM^0.66^ × FM^0.066^] is body size and sex independent, in contrast to body weight-based indexing, which demonstrates a significant inverse relationship to body weight. In the present study, RMR/FFM^0.66^FM^0.066^ increased as BMI went up, which contradicts the common view that people with higher BMI tend to have lower RMR.

The accuracy rates of our models are 75.31% and 72.22%, which are higher than those validated by Xue (70% and 62.5%) and Wang (31.5%) validated in their respective populations^19,31^. Among the pre-existing equations, the Mifflin, HB and Müller equations provided the highest percentage of accurate predictions (70.68%, 70.37% and 70.06%, respectively). However, it is noteworthy that these equations exhibited higher rates of overestimation or underestimation compared to our models. Meanwhile, we found that the selected Chinese equations provided inaccurate predictions. The accuracy rates of four selected Chinese equations were lower than 50%. The highest overestimation and underestimation rates were both higher than 60%, provided by the Xue and Singapore equation, respectively.

The observation that the prediction equation in this study explains a higher amount of variance compared to many previous studies suggests a notable improvement in the accuracy of RMR prediction. This may be explained by the fact that most of the predictive equations that have been widely used for RMR assessment are based on anthropometric variables. Multiple linear regression analysis revealed that including the duration of daily sun exposure increases the R^2^. This might suggest that daily exposure to sunlight may help explain the variation of RMR.

Daily sun exposure duration, which reflects vitamin D levels, emerged as an independent predictor of RMR in our study. This was similar to the results reported by Calton et al.^12^. Another study showed the mediating effect of vitamin D receptor gene expression in the association between 25(OH)_2_D plasma levels and RMR^34^. An increase in vitamin D levels may be associated with a decrease in RMR/kg body weight. Furthermore, it is essential to acknowledge that vitamin D supplementation also influences vitamin D levels. However, it was not included in the final model, probably due to its low consumption rate in our study.

Other demographic information we collected was not included in the final equation, probably because the frequency of some variables is low in our population. For example, participants rarely consume cigarettes, alcohol and tea compared to older people^35^. We speculate that these factors may play an important role in other populations.

To our knowledge, it is the first to take such variables into account. We have comprehensively considered various factors, including vitamin D levels, stress, physical activity, menstrual cycle, smoking, drinking, bedtime, measuring seasons and frequency of consuming caffeinated foods. The study has several limitations that should be acknowledged. First, serum 25(OH)D levels are not evaluated from blood samples. However, a study discovered that sunlight exposure is associated with serum 25(OH)D levels^36^. In addition, daily sun exposure duration is readily available. Second, the age range is narrow and older people are not taken into account. Third, some variables relied upon self-report with recall bias. Fourth, the duration of the RMR measurement was shorter (15 min). As Popp^25^ found that 87% of participants reached the most stable 5 min within the first 10 min (6–15 min) and recommended a simplified protocol that includes 5 min stabilization period, and 10 min measurement window. This is very helpful in improving compliance of participants. Future research that encompasses a broader age spectrum could provide valuable insights into how these factors interact across different age groups and contribute to RMR variations.

## Conclusions

In conclusion, the equation developed in this study exhibits a relatively better accuracy compared to previous equations, making it a promising tool for predicting RMR in young Chinese participants, especially with the inclusion of daily sun exposure duration as a predictor. The potential utility of this equation could be further enhanced through ongoing validation across diverse regions and extended to middle aged and elderly populations.

## Data Availability

The data that support the findings of this study are available upon reasonable request to the corresponding author.

## References

[1] Cuciureanu M (2023). 360-Degree perspectives on obesity. Med. Lith..

[2] Fernández Verdejo R, Galgani JE (2022). Predictive equations for energy expenditure in adult humans: From resting to free-living conditions. Obesity (Silver Spring, Md.).

[3] Amaro-Gahete F (2019). Congruent validity of resting energy expenditure predictive equations in young adults. Nutrients.

[4] Matarese LE (1997). Indirect calorimetry. J. Am. Diet. Assoc..

[5] Harris JA, Benedict FG (1918). A biometric study of human basal metabolism. Proc. Natl. Acad. Sci. U.S.A..

[6] Owen OE (1986). A reappraisal of caloric requirements in healthy women. Am. J. Clin. Nutr..

[7] Owen OE (1987). A reappraisal of the caloric requirements of men. Am. J. Clin. Nutr..

[8] Muller MJ (2004). World health organization equations have shortcomings for predicting resting energy expenditure in persons from a modern, affluent population: Generation of a new reference standard from a retrospective analysis of a German database of resting energy expenditure. Am. J. Clin. Nutr..

[9] Mifflin MD (1990). A new predictive equation for resting energy expenditure in healthy individuals. Am. J. Clin. Nutr..

[10] Mao Q (2020). Basal energy expenditure of Chinese healthy adults: Comparison of measured and predicted values. Biomed. Environ. Sci..

[11] Pavlidou E, Papadopoulou SK, Seroglou K, Giaginis C (2023). Revised Harris-benedict equation: New human resting metabolic rate equation. Metabolites.

[12] Calton EK (2016). Vitamin D status and insulin sensitivity are novel predictors of resting metabolic rate: A cross-sectional analysis in Australian adults. Eur. J. Nutr..

[13] van der Kooij MA (2020). The impact of chronic stress on energy metabolism. Mol. Cell Neurosci..

[14] Auvichayapat P (2008). Effectiveness of green tea on weight reduction in obese Thais: A randomized, controlled trial. Physiol. Behav..

[15] Careau V (2021). Energy compensation and adiposity in humans. Curr. Biol..

[16] Zitting K (2018). Human resting energy expenditure varies with circadian phase. Curr. Biol..

[17] Tanaka N (2022). Relationship between seasonal changes in food intake and energy metabolism, physical activity, and body composition in young Japanese women. Nutrients.

[18] Gould LM (2021). Impact of follicular menstrual phase on body composition measures and resting metabolism. Med. Sci. Sports Exerc..

[19] Xue J, Li S, Zhang Y, Hong P (2019). Accuracy of predictive resting-metabolic-rate equations in Chinese mainland adults. Int. J. Environ. Res. Public Health.

[20] Craig CL (2003). International physical activity questionnaire 12-country reliability and validity. Med. Sci. Sports Exerc..

[21] Cohen, S. & Williamson, G. M. *Perceived Stress in a Probability Sample of the United States, The Social Psychology of Health: Claremont Symposium on Applied Social Psychology* (1988).

[22] Petrowski K (2020). Psychometric properties of an English short version of the trier inventory for chronic stress. BMC Med. Res. Methodol..

[23] Fullmer S (2015). Evidence analysis library review of best practices for performing indirect calorimetry in healthy and non-critically III individuals. J. Acad. Nutr. Diet..

[24] Sanchez-Delgado G (2018). Reliability of resting metabolic rate measurements in young adults: Impact of methods for data analysis. Clin. Nutr..

[25] Popp CJ (2020). Evaluating steady-state resting energy expenditure using indirect calorimetry in adults with overweight and obesity. Clin. Nutr..

[26] JB W (1949). New methods for calculating metabolic rate with special reference to protein metabolism. J. Physiol..

[27] Haddad F (2023). Challenging obesity and sex based differences in resting energy expenditure using allometric modeling, a sub-study of the dietfits clinical trial. Clin. Nutr. ESPEN.

[28] Liu H, Lu Y, Chen W (1995). Predictive equations for basal metabolic rate in Chinese adults: A cross-validation study. J. Am. Diet. Assoc..

[29] Yang X (2010). Basal energy expenditure in southern Chinese healthy adults: Measurement and development of a new equation. Br. J. Nutr..

[30] Camps SG, Wang NX, Tan WS, Henry CJ (2016). Estimation of basal metabolic rate in Chinese: Are the current prediction equations applicable?. Nutr. J..

[31] Wang X (2023). Predictive equation for basal metabolic rate in normal-weight Chinese adults. Nutrients.

[32] Macena MDL, Bueno NB (2023). Letter to the editor: The use of resting energy expenditure divided by body weight (Kcal/Kg) may yield inconsistencies and should be avoided. Clin. Nutr..

[33] Heymsfield SB, Thomas DM, Bosy-Westphal A, Müller MJ (2019). The anatomy of resting energy expenditure: Body composition mechanisms. Eur. J. Clin. Nutr..

[34] Sajjadi SF, Mirzaei K, Khorrami-Nezhad L, Maghbooli Z, Keshavarz SA (2018). Vitamin D status and resting metabolic rate may modify through expression of vitamin D receptor and peroxisome proliferator-activated receptor gamma coactivator-1 alpha gene in overweight and obese adults. Ann. Nutr. Metab..

[35] Xu C (2020). Long-term tea consumption is associated with reduced risk of diabetic retinopathy: A cross-sectional survey among elderly Chinese from rural communities. J. Diabetes Res..

[36] Mansibang NMM, Yu MGY, Jimeno CA, Lantion-Ang FL (2020). Association of sunlight exposure with 25-hydroxyvitamin D levels among working urban adult filipinos. Osteoporos. Sarcopenia.

